# Unlocking the Future of Periodontal Regeneration: An Interdisciplinary Approach to Tissue Engineering and Advanced Therapeutics

**DOI:** 10.3390/biomedicines12051090

**Published:** 2024-05-14

**Authors:** Tsung-Hsi Huang, Jui-Yi Chen, Wei-Hsin Suo, Wen-Rou Shao, Chih-Ying Huang, Ming-Tse Li, Yu-Ying Li, Yuan-Hong Li, En-Lun Liang, Yu-Hsu Chen, I-Ta Lee

**Affiliations:** 1 Department of Orthopedic Surgery, Taoyuan General Hospital, Ministry of Health and Welfare, Taoyuan 330, Taiwan; fossnack@gmail.com (T.-H.H.); magister.yuhsu@gmail.com (Y.-H.C.); 2 School of Dentistry, College of Oral Medicine, Taipei Medical University, Taipei 110, Taiwan; b202110028@tmu.edu.tw (J.-Y.C.); b202110030@tmu.edu.tw (W.-H.S.); b202110092@tmu.edu.tw (W.-R.S.); b202110043@tmu.edu.tw (C.-Y.H.); b202110045@tmu.edu.tw (M.-T.L.); b202110097@tmu.edu.tw (Y.-Y.L.); b202110090@tmu.edu.tw (Y.-H.L.); b202110051@tmu.edu.tw (E.-L.L.); 3 Department of Biology and Anatomy, National Defense Medical Center, Taipei 114, Taiwan

**Keywords:** regeneration, periodontal ligament, stem cell, gene therapy, enamel, 3D printing

## Abstract

Periodontal defects present a significant challenge in dentistry, necessitating innovative solutions for comprehensive regeneration. Traditional restoration methods have inherent limitations in achieving complete and functional periodontal tissue reconstruction. Tissue engineering, a multidisciplinary approach integrating cells, biomaterials, and bioactive factors, holds tremendous promise in addressing this challenge. Central to tissue engineering strategies are scaffolds, pivotal in supporting cell behavior and orchestrating tissue regeneration. Natural and synthetic materials have been extensively explored, each offering unique advantages in terms of biocompatibility and tunable properties. The integration of growth factors and stem cells further amplifies the regenerative potential, contributing to enhanced tissue healing and functional restoration. Despite significant progress, challenges persist. Achieving the seamless integration of regenerated tissues, establishing proper vascularization, and developing biomimetic scaffolds that faithfully replicate the natural periodontal environment are ongoing research endeavors. Collaborative efforts across diverse scientific disciplines are essential to overcoming these hurdles. This comprehensive review underscores the critical need for continued research and development in tissue engineering strategies for periodontal regeneration. By addressing current challenges and fostering interdisciplinary collaborations, we can unlock the full regenerative potential, paving the way for transformative advancements in periodontal care. This research not only enhances our understanding of periodontal tissues but also offers innovative approaches that can revolutionize dental therapies, improving patient outcomes and reshaping the future of periodontal treatments.

## 1. Introduction

Periodontal disease, a persistent inflammatory condition affecting the periodontium, leads to irreversible damage in tooth attachment and surrounding bone [1]. If untreated, it progresses, causing significant loss of gingival tissue, periodontal ligament, and alveolar bone [2]. This results in compromised dental aesthetics and function, often culminating in premature tooth loss. The disease’s pathogenesis involves intricate interactions between the host’s immune response, microbial colonization of the periodontal attachment, and modifying factors like tobacco smoking and genetic predisposition [3,4]. Furthermore, periodontal disease has been linked to various systemic conditions, such as diabetes, cardiovascular disease, and rheumatoid arthritis [5,6,7]. The untreated consequences of periodontal disease are far-reaching, significantly impacting an individual’s quality of life and placing a burden on healthcare systems, with substantial economic costs. Effective periodontal therapy aims at fully restoring all elements of the periodontium to their original architecture and function. This includes the reconstruction of gingival connective tissue, cementum, alveolar bone, and the periodontal ligament (PDL). To clarify, the primary aim of this review is to systematically analyze and synthesize recent advancements and ongoing challenges in the field of periodontal regeneration through the lens of tissue engineering. By doing so, we seek to highlight how interdisciplinary approaches, particularly the integration of tissue engineering, stem cell therapy, gene therapy, and innovative scaffold designs, are crucial for overcoming existing barriers and enhancing periodontal therapy. This review intends to provide a comprehensive perspective that not only informs but also encourages further research and collaboration across various scientific disciplines, ultimately advancing our ability to effectively regenerate periodontal tissues and improve clinical outcomes. In conducting this comprehensive review, we adhered to specific selection criteria to ensure the inclusion of relevant and high-quality studies. The studies selected for inclusion were primarily chosen based on their direct relevance to the field of periodontal regeneration, encompassing advancements in tissue engineering and therapeutic innovations. We focused on peer-reviewed articles published within the last decade to ensure the inclusion of the latest research while also considering seminal works foundational to the field irrespective of their publication date. Furthermore, studies were evaluated for their scientific rigor, including clear methodologies and significant outcomes. Innovative research that introduced novel methodologies or had potential high impact on clinical practices was given priority. This approach allowed us to synthesize findings that not only reflect current advancements but also highlight the trajectory of ongoing research in periodontal tissue regeneration. Crucially, the formation of Sharpey’s fibers, embedded in newly formed cementum and alveolar bone, is vital for re-establishing proper connections between the tooth and its supporting tissues [8]. Conventional methods for treating periodontal disease have limitations, often falling short of achieving complete periodontal regeneration. Key to overcoming the limitations of traditional periodontal therapies is the utilization of advanced scaffold materials, which are foundational in the field of tissue engineering. These biomaterials are selected based on their distinct chemical properties, which ensure compatibility and minimal toxicity within the biological environment. Mechanically, they must possess adequate strength and resilience to maintain structural integrity while facilitating tissue ingrowth. Biologically, their most significant properties are osteoinductivity and osteoconductivity. Osteoinductivity refers to the ability of a material to induce progenitor cells to differentiate into bone-forming osteoblasts, thereby initiating the new bone formation process. Osteoconductivity, on the other hand, refers to the ability of a material to support the attachment, growth, and spread of cells which are crucial for the formation of new bone and soft tissues. These properties ensure that the biomaterials not only mimic but actively participate in the natural bone and tissue healing processes, thereby enhancing the effectiveness of regenerative treatments. This dual capability to support cell differentiation and provide a conducive environment for tissue regeneration makes these scaffold materials indispensable in the pursuit of restoring periodontal architecture and functionality. Recent advancements in our understanding of periodontal biology, coupled with progress in scaffolding matrices, have introduced innovative treatments. These novel approaches leverage cell and gene therapy to enhance periodontal tissue reconstruction and biomechanical integration. By exploring these cutting-edge techniques, researchers are paving the way for transformative interventions that could revolutionize periodontal care, offering hope for improved outcomes, enhanced patient well-being, and a reduced economic impact on healthcare systems. Continued research and collaborative efforts in this field are essential to unlock the full potential of these emerging therapies and improve the lives of individuals affected by periodontal disease.

## 2. Advancements in Stem Cell Therapy for Periodontal Regeneration

Contemporary research has shed light on the extraordinary versatility of postnatal stem cells, particularly those extracted from human bone marrow and dental pulp tissues. These cells are adept at differentiating into a diverse array of cell types, such as osteoblasts, odontoblasts, adipocytes, and neuron-analogous cells, showcasing their potential in regenerative medicine and tissue engineering [9]. Among these, periodontal ligament stem cells (PDLSCs) have emerged as a focal point due to their multipotent nature. They not only differentiate into cementoblast-like cells and adipocytes in vitro but also play a pivotal role in generating cementum and PDL-like structures in vivo. This is particularly noteworthy for their capacity to synthesize collagen fibers that are analogues to Sharpey’s fibers, which are essential to the structural integrity of periodontal ligaments [10]. The development of innovative models to evaluate regenerative therapies and scaffolds has underscored the critical importance of maintaining space effectively to facilitate tissue regeneration [11,12]. The use of autologous PDLSC therapy, as reported by Chen et al., has been marked by safe, and promising outcomes in treating periodontal intrabony defects have been reported, showcasing the therapeutic potential of these stem cells [13]. Similarly, the work by Takanori Iwata et al. in harvesting stem cells from third molar extractions has indicated beneficial therapeutic results, adding to the body of evidence supporting stem cell-based interventions [14]. Moreover, the application of allogeneic mesenchymal stem cells (MSCs) in periodontal repair by Caplan AI et al. presents a viable approach with a lower risk of immune rejection and reduced patient discomfort, a sentiment echoed by the findings of Nuñez et al. in their experimental study involving beagle dogs, which demonstrated the effectiveness of allogeneic MSCs in periodontal regeneration [15,16]. The clinical application of autologous PDLSCs has been particularly significant, achieving a 58% mean regeneration in acute furcation defects, highlighting the regenerative capability of these cells [17]. However, the treatment of periodontal multi-walled infra-bony defects with autologous PDLSCs seeded in deproteinized bovine bone mineral (DBBM) did not show statistically significant differences when compared to the use of DBBM alone, suggesting the need for further investigation into the optimal conditions for stem cell-mediated regeneration [13]. Conversely, the application of allogeneic stem cell therapy using canine PDLSCs in conjunction with a DBBM scaffold enriched with 10% collagen has shown promising regenerative outcomes in chronic supra-alveolar defects, with significant new cementum formation, underscoring the potential of allogeneic stem cell approaches in periodontal tissue engineering [16]. Exploring the potential of different stem cell therapies, Table 1 provides an intuitive comparison, outlining the advantages and challenges of autologous and allogeneic periodontal ligament stem cell therapies in terms of therapeutic effectiveness and feasibility.

This summary encapsulates the growing field of stem cell research in periodontal regeneration, showcasing the intricate balance between its challenges and its immense potential for breakthroughs. The ability of PDLSCs to differentiate into multiple cell types, combined with cutting-edge therapeutic approaches, presents a promising future for stem cell-driven regenerative treatments. However, the varied results seen in clinical settings underscore the need for a more detailed understanding of how stem cells interact with their surroundings and how these interactions affect their ability to regenerate tissue. As the field progresses, it is crucial for future studies to delve deeper into optimizing these regenerative techniques, harnessing the full capabilities of stem cells to transform the landscape of periodontal regeneration and pave the way for new advancements in the broader realm of regenerative medicine.

## 3. Gene Therapy Ushers in a New Age of Periodontal Renewal

Recent scientific investigations have increasingly underscored the transformative potential of gene therapy as a revolutionary approach in dentistry, particularly in the domain of periodontal tissue regeneration. This cutting-edge therapeutic strategy involves the precise delivery of specially engineered genetic material directly into the periodontal tissues. The primary objective of this innovative treatment modality is to substantially amplify the regenerative capacity of these tissues. It achieves this by significantly increasing the endogenous production of vital growth factors and differentiation factors, thereby facilitating enhanced tissue repair and regeneration [18,19]. In parallel, there has been a surging interest within the dental research community in exploring the therapeutic efficacy of various biologically active substances, including enamel matrix derivatives (EMDs), bone morphogenetic proteins (BMPs), and platelet-rich plasma (PRP). These substances are heralded for their potential in promoting the re-establishment of periodontal membranes to their native anatomical positions. The use of EMDs, BMPs, and PRP in periodontal therapy represents a confluence of biotechnology and clinical dentistry, aiming to restore the structural integrity and functional dynamics of periodontal tissues through biological means. This interdisciplinary approach not only paves the way for innovative treatments in periodontology but also holds promise for enhancing the overall efficacy of periodontal regeneration protocols, thereby contributing to the advancement of dental medicine and improving patient outcomes.

### 3.1. The Impact of EMD and Gene Therapy in Periodontal Regeneration

Research indicates that a group of proteins, predominantly amelogenins, play a significant role in stimulating the deposition of cementum and fostering the regeneration of periodontal tissues [20]. These findings are further supported by evidence suggesting that the use of EMD in conjunction with synthetic bone graft materials can lead to marked clinical improvements in the treatment of severe intrabony defects [21,22]. EMD has thus emerged as a pivotal component in the field of tissue regeneration, underscoring its potential in periodontal therapy. The application of EMD in periodontal regeneration is part of a broader spectrum of regenerative medicine, where gene therapy represents a frontier in the quest for innovative treatment modalities. Gene therapy in periodontal regeneration involves the delivery of specific genes to targeted cells in periodontal tissues to encourage the expression of proteins conducive to tissue repair and regeneration. This approach, emblematic of a new era in periodontal therapy, seeks to not only address the symptoms of periodontal disease but to fundamentally alter the underlying pathological processes. By directly manipulating the genetic and molecular pathways involved in tissue regeneration, gene therapy offers a promising avenue for achieving long-term periodontal health. The synergy between EMD and advanced techniques such as gene therapy could herald a paradigm shift in periodontal regeneration. While EMD provides a scaffold and the necessary biological signals for tissue regeneration, gene therapy could enhance these processes at a molecular level, ensuring more robust and sustained outcomes. As research in this area progresses, it is anticipated that a comprehensive understanding of the interaction between these modalities will facilitate the development of highly effective treatment strategies for periodontal disease. However, it is important to note that while the potential of gene therapy in periodontal regeneration is significant, it is imperative that such interventions are thoroughly evaluated in clinical trials to ascertain their safety, efficacy, and practical applicability in routine dental practice. Thus, the integration of EMD with groundbreaking approaches like gene therapy epitomizes the evolution of periodontal treatment towards more advanced, effective, and personalized modalities. This confluence of traditional and innovative techniques promises to significantly enhance the prospects of periodontal regeneration, offering hope to patients suffering from periodontal diseases.

### 3.2. The Impact of BMPs and Gene Therapy in Periodontal Regeneration

BMPs, particularly BMP-2 and BMP-7, have emerged as pivotal players in the field of bone regeneration, commanding significant attention for their regenerative capabilities [23]. These proteins, integral to the bone morphogenetic pathway, orchestrate a myriad of cellular processes essential for bone formation and repair. BMP-7, with its pronounced role in the advanced stages of bone development, contributes substantially to the maturation and mineralization processes that are critical for achieving structural and functional bone integrity. Its utility in periodontal regeneration is particularly relevant in the context of repairing periodontal tissues that have been compromised by disease or trauma. Conversely, BMP-2 has been spotlighted for its efficacy in fostering bone regeneration within surgically induced large furcation defects in the first and second molars of adult baboons [24]. This efficacy underscores the protein’s potent osteoinductive properties, stimulating the differentiation of progenitor cells into osteoblasts, which are crucial for bone matrix formation and subsequent mineralization. This capability positions BMP-2 as a cornerstone in periodontal therapy, especially in scenarios requiring substantial bone regeneration to re-establish periodontal support structures. The critical role of BMPs in bone remodeling further underscores their significance in periodontal regeneration [25]. Bone remodeling, a continuous process involving bone formation and resorption, is essential for maintaining bone health and integrity. In the context of periodontal regeneration, the ability of BMPs to modulate this process is paramount. By promoting bone formation while potentially mitigating excessive resorption, BMPs can help restore the balance necessary for healthy periodontal tissue architecture. Advancements in gene therapy offer a promising avenue to enhance the regenerative potential of BMPs in periodontal therapy. By targeting specific genes involved in the BMP signaling pathway, gene therapy can amplify the endogenous production of BMPs or enhance the sensitivity of target cells to these proteins. This approach not only promises to bolster the efficacy of BMP-mediated regeneration but also offers a level of precision in targeting specific defects or areas of periodontal breakdown. The integration of gene therapy with BMP-based treatments opens new horizons in periodontal regeneration, enabling more effective and personalized therapeutic strategies. In conclusion, the symbiosis between BMPs, particularly BMP-2 and BMP-7, and gene therapy represents a frontier in periodontal regeneration. The intricate mechanisms by which BMPs facilitate bone and periodontal tissue regeneration, combined with the cutting-edge potential of gene therapy to optimize these effects, offer a compelling narrative for future research and clinical applications. As our understanding of these processes deepens, so too does the potential for developing more effective treatments for periodontal disease, ultimately contributing to enhanced oral health and quality of life for patients worldwide.

### 3.3. The Impact of PRP and Gene Therapy in Periodontal Regeneration

PRP represents a forefront advancement in regenerative medicine, particularly within the domain of periodontology. This autologous preparation is derived from the patient’s own blood, undergoing a centrifugation process to concentrate platelets in a small volume of plasma [26]. The resultant PRP is a powerhouse of growth factors, including but not limited to, platelet-derived growth factor (PDGF), transforming growth factor-beta (TGF-β), vascular endothelial growth factor (VEGF), and epithelial growth factor (EGF). These growth factors are pivotal to the wound healing cascade, offering enhanced capabilities for bone healing and regeneration. This is primarily due to their roles in chemoattraction, proliferation, differentiation, and angiogenesis, which are vital for tissue repair and regeneration. The efficacy of PRP in promoting bone tissue regeneration and repair has been well documented across various studies, with evidence pointing to positive outcomes in bone regeneration applications [27,28,29,30,31]. These studies underscore the potential of PRP to significantly impact the healing process, accelerating bone formation and enhancing the quality of regenerated bone. This has profound implications for periodontal regeneration, where the restoration of lost or damaged periodontal structures is a primary objective. Incorporating gene therapy into the realm of PRP-enhanced periodontal regeneration presents a novel and exciting frontier. Gene therapy holds the potential to selectively target molecular pathways involved in periodontal healing and regeneration. By manipulating genes associated with growth factor production or the expression of specific receptors on periodontal cells, it is possible to augment the regenerative capabilities of PRP. For instance, gene therapy could be used to upregulate the expression of growth factor receptors on periodontal ligament cells or osteoblasts, thereby enhancing their response to the growth factors concentrated within PRP [32]. Alternatively, gene therapy could directly increase the production of specific growth factors within the periodontal wound site, synergistically enhancing the regenerative potential provided by PRP [33]. The integration of PRP and gene therapy in periodontal regeneration heralds a new era in the management of periodontal diseases. By leveraging the inherent healing capabilities of PRP and the precise molecular targeting afforded by gene therapy, clinicians can potentially achieve unprecedented outcomes in tissue regeneration. This approach not only promises improved clinical efficacy but also offers the prospect of reduced healing times and enhanced quality of regenerated periodontal tissues. As research in this domain progresses, it is anticipated that the combination of PRP and gene therapy will unlock new paradigms in periodontal treatment, offering patients more efficient and effective solutions for periodontal regeneration. The synergy between these advanced therapeutic modalities underscores a move towards more personalized and targeted approaches in the management of periodontal diseases, aiming to restore both function and aesthetics with minimal invasiveness and maximum efficacy.

## 4. Fiber-Guided Scaffold Innovations for Multi-Tissue Periodontal Regeneration

The exploration of tissue engineering strategies for periodontal regeneration has seen significant advancements, particularly in the reconstruction of the complex tri-phasic interface consisting of cementum, the PDL, and bone within craniofacial and musculoskeletal systems. In addition to these advanced therapeutics in periodontal regeneration, it is crucial to acknowledge the significant role of non-resorbable and bioabsorbable membranes in these procedures. These membranes are pivotal in guiding tissue regeneration effectively. Non-resorbable membranes, such as expanded polytetrafluoroethylene (e-PTFE) and titanium mesh, provide a stable and durable barrier but require subsequent removal, which can be a limitation. On the other hand, bioresorbable membranes, predominantly made from materials like collagen, offer the advantage of biodegradation, eliminating the need for a second surgery to remove the membrane. These different membrane classes contribute uniquely to periodontal regeneration by maintaining space for bone growth and preventing unwanted tissue ingrowth. A recent in vitro study by Bianchi et al. highlights the biocompatibility and supportive role of bovine pericardium membranes, a bioresorbable option, in supporting human periodontal ligament fibroblast proliferation and morphological stability, further underlining the importance of selecting appropriate membrane materials based on the clinical scenario [34].

This interface is crucial for the functional integration of tissue-engineered constructs with native tissues, especially in the context of repairing craniofacial injuries. A key focus of recent research has been the development and application of oriented scaffold channels to facilitate the formation of this tri-phasic interface. Some investigations have utilized a multi-cell therapy approach within an in vivo model aimed at overcoming the challenges associated with engineering bone–ligament interfaces. The intricacies of reconstructing the cementum–PDL–bone interface are manifold, requiring the precise orchestration of tissue maturation and biomechanical properties. The utility of polarized anchoring fibers, oriented towards a mineralizing surface, has been identified as a critical factor in promoting tissue integration and functionality. Research efforts have encompassed a range of strategies, including complex reconstruction efforts [35], the application of topographical designs [36], and the cultivation of conducive microenvironments [37]. Among these, the deployment of a tissue-guiding scaffold, characterized by its specialized architecture and surface topography, has emerged as a particularly promising approach [38]. Such scaffolds have demonstrated significant potential in facilitating the regeneration of tooth–ligament–bone complexes within surgically created defect models [39]. The investigation yielded three principal findings, underscoring the efficacy of the employed strategies. Firstly, the adaptation of designed 3D scaffolds to periodontal defects was assessed, revealing a high degree of conformity (adaptation ratio = 0.962). These fiber-guiding scaffolds, with their customized architecture and micro-groove surface topography, exemplify the tailored approach necessary for effective tissue engineering [40]. Secondly, the examination of growth patterns in mineralized tissues through Micro-CT imaging delineated the advantages of fiber-guiding scaffolds in promoting tooth-supporting tissue formation in contrast to random–porous scaffolds. The quantitative analysis provided empirical support for this observation, confirming the enhanced formation of tooth-supporting tissues in groups utilizing fiber-guiding scaffolds [41]. Thirdly, the organization and maturation of ligament tissue were evaluated using histological and immunofluorescence techniques. Here, fiber-guiding scaffolds demonstrated superior periostin expression, indicative of improved ligament functionality and maturity in comparison to their random counterparts. The pursuit of regenerating ligament–bone complexes, particularly the dentin–PDL–bone complex, remains fraught with challenges [42]. However, innovative approaches leveraging oriented 3D pores and tissue compartmentalization have shown promise in regenerating functional ligament tissue within osseous defect models [43]. The strategic control over cell and tissue compartmentalization is pivotal for the synchronized regeneration of multiple tissue types [44]. The carefully designed scaffold topography has been instrumental in enhancing the formation, organization, and functionality of these multi-tissue interfaces [45]. In summary, the advancements in scaffold design and compartmentalization underscored by this research hold significant promise for the field of craniofacial and musculoskeletal tissue interface reconstruction. The biomimetic architecture of these scaffolds plays a critical role in the creation of functional ligamentous interfaces and distinct tissue regions, highlighting a pivotal advancement in periodontal regeneration strategies. This comprehensive review underscores the critical importance of scaffold design, topography, and compartmentalization in advancing the field of tissue engineering for periodontal regeneration.

## 5. Injectable Calcium Phosphate Cement (CPC) for Periodontal Regeneration in Dogs

Within the domain of periodontal regeneration, the application of injectable CPC has emerged as a noteworthy innovation, particularly in the context of canine models. This material has been recognized for its capacity to foster stable bone formation and facilitate the healing of periodontal tissues, thereby offering a viable alternative to traditional grafting techniques. The significance of this advancement lies not only in its efficacy but also in addressing the limitations associated with autologous transplantation procedures. These limitations include the scarcity of graft materials and the invasive nature of surgeries that require tissue harvesting from intraoral or extraoral sites. Moreover, while allografts have been explored for their osteoinductive or osteoconductive potentials, their performance has been marred by inconsistencies in outcomes [46,47,48,49]. To further elucidate the potential of CPC in periodontal tissue regeneration, a controlled study was undertaken involving six healthy adult beagle dogs. These dogs were subjected to induced periodontitis through the placement of stainless steel mesh on the mesial side of their maxillary canines, creating a standardized intrabony defect model. These defects were subsequently treated with CPC, whereas non-grafted defects were maintained as controls to provide a comparative baseline for healing outcomes. Following a twelve-week post-operative period, a detailed evaluation encompassing histological and histometric analyses was conducted to ascertain the extent of periodontal tissue healing. Clinical observations post-procedure indicated a smooth healing process across all twelve treated sites, characterized by minimal inflammatory responses. Notably, the period following surgery did not witness any adverse reactions such as material exposure, infection, or suppuration, underscoring the biocompatibility of CPC. An initial inflammatory response was recorded immediately post-surgery; however, it was less pronounced in the experimental sites compared to the control sites. Histological assessments further reinforced the therapeutic potential of CPC, highlighting the absence of acute inflammatory responses or foreign body giant cell reactions in the treated specimens [50]. A significant finding was the pronounced formation of new bone and cementum within the experimental sites, with the height of newly formed bone closely mirroring that of the intact host bone along the root surface. This observation suggests the effective integration of CPC with the host bone, particularly noted at distal and apical regions of the cement. Importantly, the analysis did not reveal any instances of root resorption or ankylosis adjacent to the CPC-treated areas, pointing to the material’s safety and efficacy in supporting periodontal regeneration. The insights garnered from this study illuminate the promising role of injectable CPC in advancing periodontal tissue engineering strategies. By offering a scaffold that supports the regeneration of periodontal defects in a canine model, CPC stands as a pivotal tool in the repertoire of regenerative dentistry, showcasing a significant step forward in the pursuit of optimal periodontal healing and reconstruction methodologies.

## 6. Alginate–Fibrinogen-Infused CPC for Advanced Bone Repair

In the realm of maxillofacial surgery, addressing bone injuries stemming from accidental damage presents significant challenges. Traditional methods, often reliant on non-living materials, are increasingly being supplanted by cell-based tissue engineering approaches, which offer a more dynamic solution to bone repair [51]. Given that calcium phosphate is a predominant component of hard tissues, its derivative, CPC, emerges as a logical choice for bone repair applications [52]. However, the innovation does not stop at the selection of material; the method of cell delivery within these constructs is critical. The ideal material must not only protect the encapsulated cells but also facilitate rapid biodegradation to accommodate new tissue growth. In this context, alginate and fibrin emerge as exemplary materials due to their biocompatibility and functional properties. Alginate, a well-documented hydrated gel, offers a biocompatible matrix for cell encapsulation [53], while fibrin, a key player in blood clotting, provides structural support and aids in cell adhesion and migration [54]. Leveraging these properties, the development of alginate–fibrin hydrogel microfibers (Alg-Fb MF) represents a significant advancement in cell delivery technologies. These microfibers have been engineered to optimize cell viability and release, with experiments indicating that varying fibrinogen concentrations can affect the microfiber’s physical properties and degradation rates. Specifically, the incorporation of fibrinogen was found to increase the diameter of alginate microfibers, with higher concentrations leading to less degradability but potentially more structural support for encapsulated cells. Human bone marrow mesenchymal stem cells (HBMSCs) encapsulated within Alg-Fb MF demonstrate remarkable viability, highlighting the potential of this composite material for bone tissue engineering. The Alg-0.4% Fb MF composition, in particular, showed optimal performance in terms of cell release and proliferation, with cells being released as early as day 2 post-encapsulation and exhibiting significant proliferation rates [55]. The research further explores the application of Alg-Fb MF in an injectable stem cell delivery system specifically designed for bone tissue engineering. This innovative system employs alginate–fibrinogen microfibers for encapsulating HBMSCs, thereby ensuring cell protection during the delivery process and enhancing cell viability, migration, and differentiation post-delivery. When applied to a mandibular bone defect model, the CPC-MF-HBMSCs treatment group exhibited a threefold increase in new bone formation compared to controls lacking cell delivery. The Alg-Fb MF microfibers not only facilitated cell spreading and migration but also promoted the self-assembly of cells into functional units conducive to bone regeneration. This injectable cell delivery system, with its combination of alginate–fibrin hydrogel microfibers and calcium phosphate cement, offers a promising avenue for enhancing bone regeneration across dental, craniofacial, and orthopedic applications. By integrating cell-protective materials with effective delivery mechanisms, this approach represents a significant leap forward in the field of periodontal regeneration, underscoring the importance of material science and bioengineering in advancing tissue engineering strategies.

## 7. Innovative Biological Framework for Developing the Periodontal Complex through Multiple Phases

The domain of periodontal regeneration has witnessed a notable evolution with the advent of cell sheet engineering, particularly through the application of thermally responsive polymers. This innovative approach leverages the unique properties of poly(N-isopropylacrylamide) (PIPAAm), which is grafted onto cell culture plates [56], to cultivate cell-rich sheets. These sheets, laden with PDL stem cells, represent a significant stride forward in the regeneration of damaged periodontal tissues. When combined with osteoconductive materials like β-tricalcium phosphate (β-TCP) [57], this methodology facilitates the orchestrated regeneration of the complex periodontal structure, including the cementum, PDL, and bone. To augment the capabilities of periodontal regeneration further, the integration of PDL cell sheets with poly-ε-caprolactone (PCL) scaffolds has been explored. This research introduces biphasic scaffolds that synergize PCL nanofibrous scaffolds with hydroxyapatite (HA)/PCL scaffolds created through Fused Deposition Modeling (FDM). This combination not only stabilizes PDL cell sheets upon the dentin surface but also encourages the attachment of fibrous connective tissues, bridging the newly formed PDL and bone. Such compartmentalized constructs mimic the natural periodontal structure on a micron scale, enhancing the hierarchical regeneration of periodontal tissues [58]. Innovative preclinical studies have ventured into osteoconductive biphasic scaffolds which integrate calcium phosphate (CaP) with PCL nanofibrous scaffolds for the treatment of periodontal fenestration defects. Modifications to the electrospun PCL membrane to increase hydrophilicity and a CaP coating were introduced to bolster its osteogenic potential. These CaP-PCL membranes play a pivotal role in the delivery and securement of PDL cell sheets to the root-exposed periodontal defects, catalyzing both cementogenesis and bone formation, thus promoting periodontal attachment [59]. The amalgamation of cell sheet engineering with scaffold integration emerges as a promising frontier in the field of periodontal tissue regeneration. Utilizing PDL cell sheets in tandem with an array of scaffold materials and their subsequent modifications presents a versatile and bioactive strategy primed for clinical application in the realm of effective periodontal regeneration.

## 8. Advancements in 3D Printing for PDL Regeneration

The burgeoning field of dental regeneration is witnessing a significant shift towards the application of 3D printing technologies aimed at the precise replication of the complex hierarchical structures inherent to periodontal tissues. This innovative approach heralds a new era in the design and fabrication of scaffolds, granting unparalleled control over their dimensional and architectural attributes to closely emulate the natural environment of PDL tissues [60]. The adoption of 3D printing and bioprinting technologies for the creation of dental biomimetic constructs is predominantly centered around methodologies such as Fused Deposition Modeling (FDM), Direct Ink Writing (DIW), and Selective Laser Melting (SLM), each offering unique advantages in the pursuit of tissue engineering [61]. FDM has been instrumental in fabricating scaffolds that mimic the extracellular matrix (ECM), bone, and cartilage, providing a conducive framework for the development of functional tissues. These scaffolds support critical biological processes, including cellular attachment, proliferation, and differentiation, essential for the regeneration of periodontal tissues [62]. Moreover, extrusion-based bioprinting technologies have advanced to produce collagen-based microfibers in both straight and waveform patterns. This advancement is pivotal for guiding the growth and organization of PDL cells, showcasing the potential for enhanced tissue integration and functionality [63]. DIW plays a crucial role not only in assessing the printability of dental bioinks, but also in facilitating the fabrication of two-dimensional structures with precise thickness, further enriching the arsenal of tools available for dental tissue engineering [61]. The sphere of 3D biofabrication has also expanded to include a variety of hydrogels, such as fibrin, collagen, alginate, and gelatin methacryloyl (GelMA), each selected for their biocompatibility and ability to support cellular activities conducive to tissue regeneration [64]. SLM emerges as a cutting-edge technique for the curing of GelMA hydrogels laden with cells. It allows for meticulous control over scaffold architecture, enabling the creation of structures with porous channels that not only support nutrient supply but also facilitate cell growth and differentiation. GelMA hydrogels, modified with methacrylic anhydride to introduce methacryloyl groups, undergo photopolymerization under UV light to form a crosslinked network. This process yields a hydrogel with customizable mechanical properties and geometric shapes, ideal for repairing irregular bone defects and closely mimicking the three-dimensional microenvironment of the natural ECM [65]. The advent of PDL 3D printing represents a transformative advancement in the field of periodontal regeneration. By faithfully replicating the native structure of the PDL, these 3D-printed constructs promise to significantly enhance the regeneration of periodontal tissues, facilitate proper tooth alignment, and bolster the long-term stability of dental implants.

## 9. Conclusions

The rapid advancements in tissue engineering and regenerative medicine have provided unprecedented strategies for treating periodontal tissues damaged by disease or trauma. This interdisciplinary approach, combining stem cell therapy, gene therapy, innovative scaffold designs, and 3D printing technologies, has significantly enhanced our ability to mimic the complex architecture and functionality of the periodontium. Particularly, breakthroughs in 3D printing technology have made it possible to precisely replicate the intricate structures of periodontal tissues, aiding in the accurate reconstruction of the periodontal ligament and supporting the formation of a functional interface between the tooth and surrounding bone. Despite these advancements, challenges remain in achieving complete and seamless tissue integration, vascularization, and the development of biomimetic scaffolds that fully replicate the natural periodontal environment. The future of periodontal regeneration will focus on the continuous improvement of these technologies and strategies, overcoming current limitations through innovative research and interdisciplinary collaboration. By harnessing the full potential of tissue engineering and regenerative medicine, we can look forward to developing more effective, efficient, and minimally invasive treatments for periodontal diseases. This not only promises to improve quality of life for patients but also provides insights and methodologies that can be applied to the regeneration of other complex tissues and organs.

## Figures and Tables

**Table 1:** Evaluating autologous versus allogeneic periodontal ligament stem cell therapies.

Periodontal Ligament Stem Cell Therapy			
		Autologous	Allogenic
**Advantages**		No immune rejection response, demonstrating good therapeutic efficacy	Allogeneic stem cells can be readily acquired
**Disadvantages**		Extracting stem cells necessitates the removal of a tooth	The compatibility and efficacy of this approach remain subjects of ongoing research

## Data Availability

The data presented in this study are available in this article.

## Abbreviations

PDL: periodontal ligament; EMDs: enamel matrix derivatives; BMPs: bone morphogenetic proteins; PRP: platelet-rich plasma; PDGF: platelet-derived growth factor; TGF-β: transforming growth factor-β; VEGF: vascular endothelial growth factor; EGF: epithelial growth factor; MSCs: mesenchymal stem cells; PDLSCs: periodontal ligament stem cells; DBBM: deproteinized bovine bone mineral; CPC: calcium phosphate cement; Alg-Fb MF: alginate–fibrinogen microfibers; HBMSCs: human bone marrow mesenchymal stem cells; PIPAAm: poly(N-isopropylacrylamide); β-TCP: β-tricalcium phosphate; PCL: poly-ε-caprolactone; HA: hydroxyapatite; FDM: Fused Deposition Modeling; DIW: Direct Ink Writing; SLM: Selective Laser Melting; GelMA: gelatin methacryloyl; ECM: extracellular matrix.
